# Resistance of the fiber-derived geotextile from *Typha domingensis* submitted to field degradation

**DOI:** 10.1038/s41598-024-56978-3

**Published:** 2024-04-15

**Authors:** Francisco Sandro Rodrigues Holanda, Luiz Diego Vidal Santos, Jeangela Carla Rodrigues De Melo, Gizelio Menezes Boge, Eliana Midori Sussuchi, Brenno Lima Nascimento, Marcos Vinícius Quirino dos Santos, Marla Ibrahim Uehbe de Oliveira

**Affiliations:** 1 Agronomy Engineering Department, Universidade Federal de Sergipe-UFS, São Cristóvão, Sergipe Brazil; 2 Graduate Program in Intellectual Property Science, Universidade Federal de Sergipe-UFS, Avenida Marechal Rondon Jardim S/N - Rosa Elze, São Cristóvão, Sergipe 49100-000 Brazil; 3 Chemical Engineering Department, Universidade Federal de Sergipe-UFS, São Cristóvão, Sergipe Brazil; 4 Graduate Program in Materials Science and Engineering (P2CEM), Universidade Federal de Sergipe-UFS, São Cristóvão, Sergipe Brazil; 5 Chemistry Department, Universidade Federal de Sergipe-UFS, São Cristóvão, Sergipe Brazil; 6 Biological Sciences Department, Universidade Federal de Sergipe-UFS, São Cristóvão, Sergipe Brazil

**Keywords:** Tensile strength, Soil bioengineering, Natural fiber, Ecology, Natural hazards, Engineering, Materials science

## Abstract

Geotextiles made from plant fibers creates a suitable environment for plant growth as part of soil bioengineering techniques. The faster decomposition of plant fiber geotextiles compared to synthetic ones demands the use of composites that enhance their waterproofing and extend their durability in the environment. The objective of this work was to evaluate the resistance of a geotextile made with *Thypha domingensis* to degradation caused by climatic variables. Tensile strength tests were conducted in the laboratory in order to evaluate the degradation of geotextiles treated with single and double layers of waterproofing resin. Based on Scanning Electron Microscopy (SEM) images, it was verified that applying double layer of waterproofing resin delays the fibers degradation up to 120 days of exposure to the effects of climatic variables other than temperature. The maximum resistance losses due to the geotextile's exposure to degradation were statistically significant for all three treatments: control-without waterproofing resin, with one layer resin, and with two layers resin. Therefore, waterproofing resin, provides a long-term protective solution for geotextiles made from cattail fibers.

## Introduction

Soil erosion is a geomorphic process resulting from soil disruption, transport of particles, rock fragments, organic matter, and siltation of water systems^1^. Features of topography, geomorphology, vegetation, and anthropic activities are factors that interfere on the stability of slopes and is associated to erosion processes^2^. Mitigating measures to control erosion on slopes use different techniques, such as soil bioengineering^3,4^. Soil bioengineering brings together materials and techniques from the field of biology and civil engineering based on the use of inert materials such as steel, wood, rock, and concrete, as well as biological materials such as live cuttings and seedlings of plant species, and also geotextiles^5,6^. Also soil bioengineering involves techniques that use plants as living building materials to control soil erosion^7^, torrential floods and landslides and in order to reach ecological restoration or even the reintroduction of species in degraded lands^8^, offering a range of benefits for humans and nature.

The utilization of natural geotextiles in conjunction with sustainable technologies has demonstrated improvement in soil protection, while also proving to be more economically viable than traditional engineering techniques^9^. Geotextiles provide direct protection against soil erosion, exhibiting controlled degradation, and help in the process of revegetation, minimizing erosion processes^10^.

Geosynthetics, such as geotextiles and geomembranes, designed according to the geotechnical land´s needs has become an emerging field in civil engineering and other fields in which they offer varied areas of application^11^. The main advantages of synthetic products are their slow biodegradability, which leads to greater durability, and their adaptability for specific engineering uses at a reduced cost^12^. However, low tensile modulus, ease of photodegradation, lower permeability, and restriction to the development of soil flora and fauna are among the main disadvantages of geosynthetics for the protection and recovery of degraded areas^13^.

The use of geotextiles offers rapid protection against erosive processes in the soil, increases the resistance of the slopes, and promotes the rapid growth of vegetation, all of which are very effective in controlling erosion^14^. According to Yan et al^15^., by allowing the development of a vegetation cover, geotextiles create an environment that intercepts the precipitation and reduces splashes due to the different morphologies and structures of the developed vegetation, ultimately controlling soil erosion.

Most of these geotextiles originate from synthetic materials can fail in the field due to detachment or inadequate interaction between the soil and the geosynthetic, which leads to loss of effectiveness^16^. Additionally, the use of geosynthetics can generate environmental issues such as soil pollution and the accumulation of microplastics^17^. Thus, it is necessary to choose a product that is efficient and ecologically stable in its long-term properties^18^.

Vegetable fibers used in geotextile production offer a favorable combination of mechanical and environmental properties ^19^. These geotextiles present advantages like simplified implementation, compatibility with the surrounding landscape and environment, contributing to the preservation of local biodiversity^20^. Therefore, comprehensive studies on the durability of natural geotextiles, both in laboratory and field settings, are important to better recognize their properties and ensure successful slope and riverbank stabilization, promoting soil anchorage^21^.

Some natural geotextiles made from *Cocos nucifera* L., *Agave sisalana* Perrine ex Engelm, and *Hibiscus canabinus* L. fibers have good mechanical properties comparable to synthetic fabrics for flooring and, therefore, may be advantageous for soil bioengineering applications^22^.

Another species that presents outstanding potential for the manufacture of geotextiles from its fibers is *Thypha domingensis*, a plant with long green leaves that grows on the banks of lakes and dams, marshes and swamps and can be found throughout tropical and subtropical America, being used for the manufacture of handicrafts^23^.

*T. domingensis*, a plant species extensively distributed across the tropical and subtropical regions of the Americas, is commonly found in lake margins, marshes, and wetland areas^24^. Its widespread availability, coupled with a relatively straightforward process for fiber collection and processing, as well as its ease of cultivation, render it a viable candidate for the production of natural geotextiles^25^. Furthermore, as a renewable and biodegradable resource, geotextiles derived from *T. domingensis* are environmentally sustainable and can be utilized in soil stabilization projects without any adverse impacts on the environment^26^.

Regularly found in wetland ecosystems, its extensive presence in over 80 countries makes it a suitable resource to produce geotextiles on a large scale^27^. In tropical countries such as Brazil, Mexico, and India, *Typha domingensis* is notably abundant, often classified as an invasive species due to its rapid growth^28^.

Like geosynthetics, geotextiles made from natural fibers suffer deterioration from different environmental factors, including friction, moisture, and exposure to UV light. Geotextiles exposed to UV light at wavelengths between 300 and 400 nm are more vulnerable to photo-oxidation ^29^. In geosynthetics, to avoid its degradation in long-term uses, supplemental additives help the fibers to remain stable for longer^30^.

The most common protective additives applied in geosynthetics include *Chimassorb 944* and *carbon black,* which helps polymers to resist the UV light damage that accelerates material degradation^31^. However, little has been explored on the geotextiles manufacture^32^, related to the development of protective compounds in order to increase the durability of natural fiber geotextiles in the field, and on the complexities of the effects of resistance and durability from the interaction with the environmental variables and the soil, since the mechanisms are commonly measured based on empirical procedures. Although additives delay the effects of degradation, the decomposition of the fibers still happens, with the long-term durability of the product being affected^33^, thus, it is necessary to verify the durability of the geotextiles both in the field and in laboratory tests to better understand fiber-composite interactions, helping the slope stabilization and potential floristic recovery^34^.

Therefore, to contribute to the research on the durability of geotextiles composed of natural fibers, the objective of this work was to evaluate the resistance of a geotextile made with *T. domingensis* to degradation caused by climatic variables.

## Materials and methods

### Plant collection and processing

**Figure 1:**
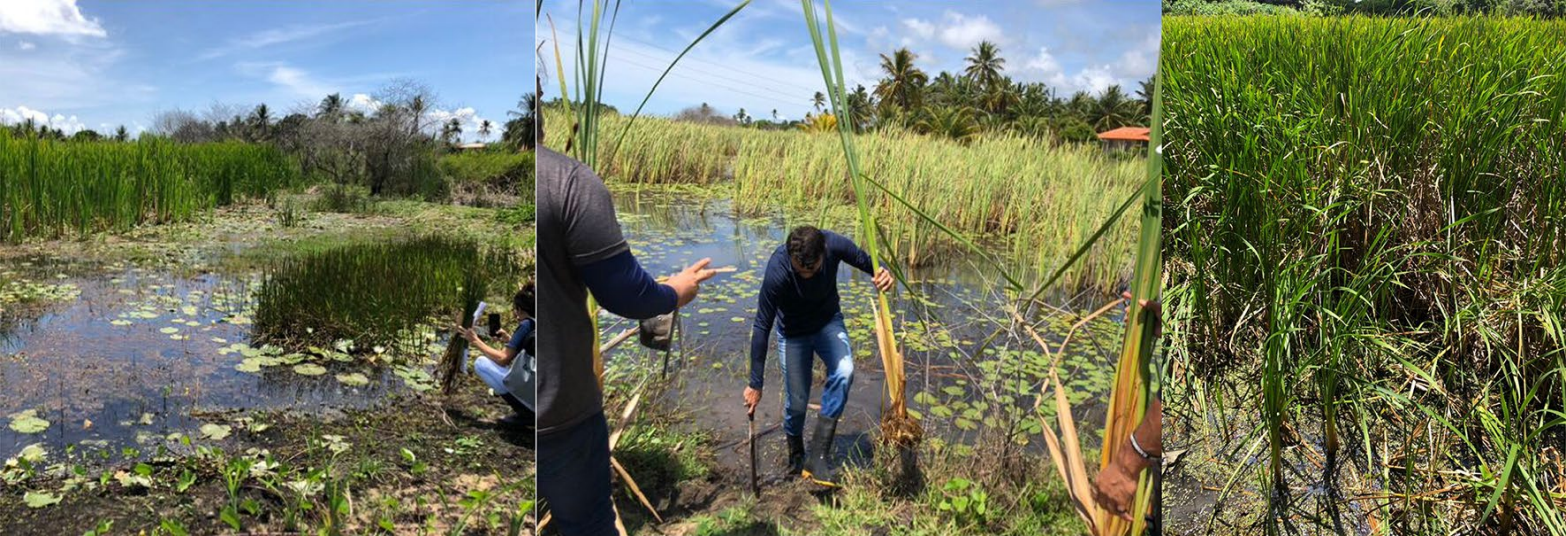
Harvesting of *T. domingensis* for fiber extraction and geotextile production.

Samples of *T. domingensis* (Fig. 1) plants were selected and collected based on the mechanical application of the fiber, cellulosic and lignin content reported in the literature, in 2 (two) municipalities of the Lower São Francisco River stretch (Northeastern Brazil), between 2019 and 2020. *T. domingensis* is a perennial herbaceous plant of the genus Typha, of the Typhaceae family included in the SisGen database.

After collection, the samples were taken to the laboratory for drying, fraying and separation of the fibers in order to manufacture the geotextiles. The studied species were registered in the Sistema Nacional de Gestão do Patrimônio Genético e do Conhecimento Tradicional Associado (National System for the Management of Genetic Heritage and Associated Traditional Knowledge) with the following registration code SisGen A2B3842.

Obligatory licenses were obtained for the collection of wild plant specimens in Brazil, and the collection protocol was carried out in accordance with the standard procedures recommended by the National System for the Management of Genetic Heritage and Associated Traditional Knowledge “SisGen”. The formal identification of the plant material was made by Dr. Maria Luiza Silveira de Carvalho, professor of Botany at Biology Institute-Universidade Federal da Bahia. Voucher samples were kept in the Herbarium of Universidade Federal de Sergipe (ASE), São Cristóvão-SE, Brazil.

### Experiment implementation

This study was carried out on a steep slope of 18.3° in a soil classified as a Typic Quartzipsamment (10°55′47.2″ S 37°06′12.6″ W), with more than 90% of sand particles in the texture, and very low water retention capacity, strongly influencing in the geotextile degradation rhythm, exposed to climatic variables, from march to august 2021, during the rainy season, with the temperature varying from 21 °C to 29 °C, and total rainfall in the period of 720 mm, located on the Campus of the Universidade Federal de Sergipe, Municipality of São Cristóvão, Sergipe state, in northeastern Brazil. Geotextiles made with *T. domingensis* fibers measuring 1 m^2^ were randomly distributed and submitted to the following treatments: (a) geotextile with no waterproofing resin (control); (b) geotextile treated with a single layer of waterproofing resin; and (c) geotextile treated with a double layer of waterproofing resin.

### Production of geotextiles

The production process of the *T. domingensis* geotextile involves carefully planned steps to ensure the quality and effectiveness of the material. Initially, the plant was harvested using appropriate cutting tools to preserve the integrity of the fibers, which is necessary to keep the strength of the geotextile. After harvesting, the fibers was dried in the shade for about six to eight days, and storaged in dried environment.

The thickness and fibers length, which may vary depending on environmental conditions, were meticulously selected in the manufacturing process of the geotextile. The weaving process involves twisting and braiding the fibers on a wooden mold, to provide resistance to traction.

### Waterproofing resin treatment

The geotextiles were manufactured from *T. domingensis* fibers and subjected to treatment with a colorless wood waterproofing resin by Hydronorth©. The selection of a colorless wood waterproofing resin in the geotextile´s production was driven by its specific properties that align with the desired characteristics of the product^35^. This type of resin provides robust waterproofing capabilities without the addition of harmful chemicals or heavy pigments that could leach into the environment^36^, thus preserving the biodegradability and ecological compatibility of the geotextile. Additionally, this product was compatible to the available apparatus used to spray over the fibers.

The geotextiles were covered with single and double layers of waterproofing resin and then exposed to the effect of climatic variables degradation on the slope, such as rain, solar radiation, temperature, and wind. The waterproofed material was kept for incubation for approximately 48 h at room temperature before field exposure^37^. This time length guaranteed suitable fixation and effectiveness of the waterproofing treatment.

Geotextile samples measuring 30 cm in length were collected every 30 days over a period of 180 days. The environmental conditions (temperature, humidity, precipitation, and solar radiation) were monitored daily through the gathering of information conducted by the meteorological station^38^.

### Natural degradation of geotextiles and scanning electron microscopy analysis

The natural degradation test analyzed the behavior of the geotextile under rain, solar radiation, temperature, wind, etc. and was carried out at different levels of degradation in the field. Six sample collections were performed at 30 days intervals as follows: T_1_ (30 days), (T_2_ 60 days), (T_3_ 90 days), (T_4_ 120 days), (T_5_ 150 days) and (T_6_ 180 days) of exposure.

Scanning Electron Microscopy (SEM) images were obtained through randomly selected samples at predefined sampling times, and fibers were cut into 1 cm × 1 cm samples for evaluation. The samples were metalized with gold in the Cressington of the manufacturer Kurt J. Lesker 108 of the Laboratory of Corrosion and Nanotechnology (LCNT). The used equipment was a HITACHI scanning electron microscope, model TM 3000, from the Center for Multiuser Chemistry Laboratories (CLQM-UFS), and the images were obtained on the surface of the metallized sample through the scanning of an electron beam of 15 keV and under vacuum^39^.

### Mechanical tests on tensile strength

**Figure 2:**
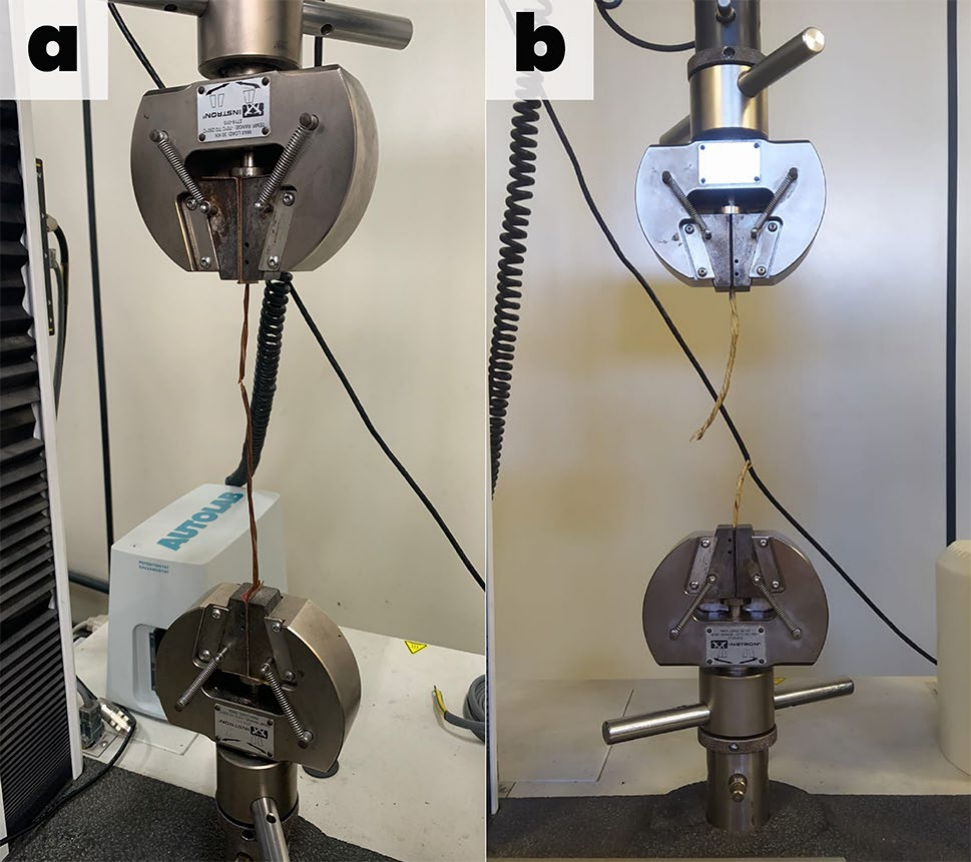
Tensile strength tests of *T. domingensis* fibers.

Unconfined tensile strength tests were carried out at the Materials Engineering Laboratory of the Universidade Federal de Sergipe (UFS) using an EMIC Model DL universal testing machine with a maximum capacity of 300 KN^40^. In order to conduct the tensile strength tests of the geotextile fibers, 7 (seven) samples were considered after the field degradation processes. The applied distance between the steel jaws with the inner faces was of 100 mm (Fig. 2), and the deformation speed was of 20 mm/min.

To secure the fibers in the universal testing machine, a pair of steel claws was used, with the internal faces contacting the fibers to prevent a possible displacement during the application of traction forces. Load VS curves were performed within the test’s proceeeding. The analyzed characteristics were tensile strength at maximum load (α max), tensile deformation (ε^ii)^ and secant stiffness (J_sec_).

### Statistical data analysis

In order to evaluate the interaction between the different treatments and the physical characteristics of the fibers throughout the evaluation period (T_1_—30th day to T _7_—180th day), multivariate analysis of variance for repeated measures (MANOVA-MR) were performed. A regression analysis was also performed to investigate the effect of time on degradation in the mechanical characteristics of resistance of *T. domingensis* fiber that differed with the application of two levels of waterproofing resin.

Post hoc analyses for main effects and interaction effects (exposure times*waterproofing resin) were performed using the Bonferroni test and considering the effect size (*r*) by Cohen’s D^41^ and F-value (*F*).

To describe the statistical strength of the physical resistance of the geotextile with an application of waterproofing resin, the effect sizes were evaluated by Eta squared (η^2) and Cohen's D were used for analyzes means and differences in means (ΔM). The effect size refers to the power that an independent variable (VI) exerts on a dependent variable (DV). In this study, the size of the obtained effect by the VD characterizes the effect of the geotextile treatment in relation to the control treatments (T_1_ and without waterproofing resin).

The data normality was assessed through the Kolmogorov‒Smirnov (KS) and Shapiro‒Wilk (SW) residual tests. The variance homogeneity assumption was evaluated through the Levene’s test. The assumption of sphericity was obtained by the Mauchly test, and when not complied with, Greenhouse‒Geisser values for sphericity < 0.75 and Huynh–Feldt for sphericity > 0.75 will be assigned.

Bootstrapping procedures with 1000 resamplings; 95% Bias-Corrected and Accelerated (BCa) Confidence Interval (CI) were implemented to achieve a greater reliability of the results, to correct deviations from normality in the sample distribution and differences between the sizes of the groups, and to present a confidence interval of 95% for differences among the average data^42^. All statistical tests were performed at *p value* = 0.05, and mean differences (**ΔM)** and marginal estimates were performed with confidence interval adjustment throughout the Bonferroni method.

Regression assessments were applied to investigate to what extent the waterproofing agent applied on the fibers under different levels of climatic degradation explains the longevity of its resistance through the physical studied characteristics.

## Results and discussion

### Tensile strength at maximum load (α max)

During the samples’ evaluation, the tensile strength was gradually reduced under the effect of climatic variables, promoting the geotextile degradation over time. The normality distribution data of the residuals showed that only treatment T_1(30 days)_ [KS = 0.263, *p* < 0.001; SW = 0.821, *p* < 0.001] did not show a normal distribution, and Levene’s test showed that all groups showed homogeneity of variance. Mauchly's sphericity test did not meet the sphericity assumption (Mauchly's *W* = 0.002 c^2^ (20) = 98.947, *p* = 0.001),R and the general result of ANOVA-MR (Since the sphericity test was not conducted, Huynh-Feldt values (> 0.75) were considered for within-subjects effects) showed that there were statistically significant differences in tensile strength over the 180-day experiment (F (4, 62.908) = 9.013, *p* < 0.001; *η*^2^  = 0.255).

**Figure 3:**
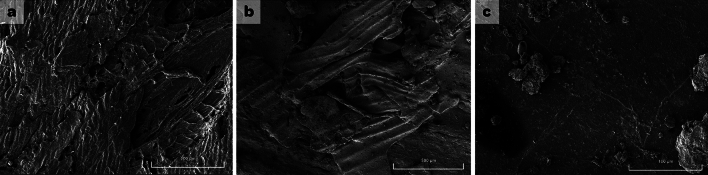
SEM images of *T. domingensis* fibers after 180 days without treatment (**a**), one layer of waterproofing resin (**b**), and two layers of waterproofing resin (**c**).

The results showed that when exposed to natural degradation, *T. domingensis* fibers decreases in tensile strength at maximum load throughout the aging process of 180 days. A statistically significant difference was presented by the samples without waterproofing resin (*F* (1, 45) = 31,386, *p* < 0.032; *R*^2^ = 0.411), marginally significant for the samples with the application of a layer of waterproofing resin (single layer) (*F* (1, 45) = 22,618, *p* < 0.045; *R*^2^ = 0.682) and not significant for double layer (*F* (1, 47) = 23,853, *R*^2^ = 0.752, *p* < 0.065). These data demonstrate that the maximum tensile strength of the fibers has low variability over a 180-day exposure to natural degradation but vary with or without waterproofing resin treatments, showing slightly higher in those that received protective treatment with waterproofing resin than the ones without the application of waterproofing resin. Figure 3 shows the behavior on Scanning Electron Microscopy (SEM) images related to treatment with and without waterproofing resin.

The images show the SEM results of the geotextile samples that were collected after a 180-day exposure to degradation. The geotextile sample without waterproofing treatment (Fig. 3a) demonstrates that a complex structure of the fiber presents cellulosic loss points of 10.153 µm diameter and 1.581 µm in diameter, as well as other minor degradation points. On the samples of geotextile fibers treated with waterproofing resin with one layer (Fig. 3b), the presence of longitudinal cracks could be noted, even though there were no transverse cracks. Fibers treated with two layers of waterproofing resin (double layer) (Fig. 3c) showed greater material integrity, and fiber breakage, degradation points, or cracks were not observed.

A study performed by Carneiro et al^43^. using SEM to evaluate the degradation behavior in manufactured geotextiles showed that during exposure to natural light, geotextiles present extensive damage to natural UV light exposure, with many fibers cracked and broken. It is important to note that the previous authors evaluated geotextiles treated with protective stabilizers in their compositions, evaluating damage to geotextiles using SEM techniques. Franco et al^33^., analyzing the behavior of geotextiles observed that when not treated with UV stabilizers, they present degraded points in a shorter time than those treated with stabilizers. Then it is possible to note that in the absence of any stabilizers, geotextiles are highly susceptible to UV degradation, which explains the observed damage in Fig. 2a and the loss of physical resistance of the material.

**Figure 4:**
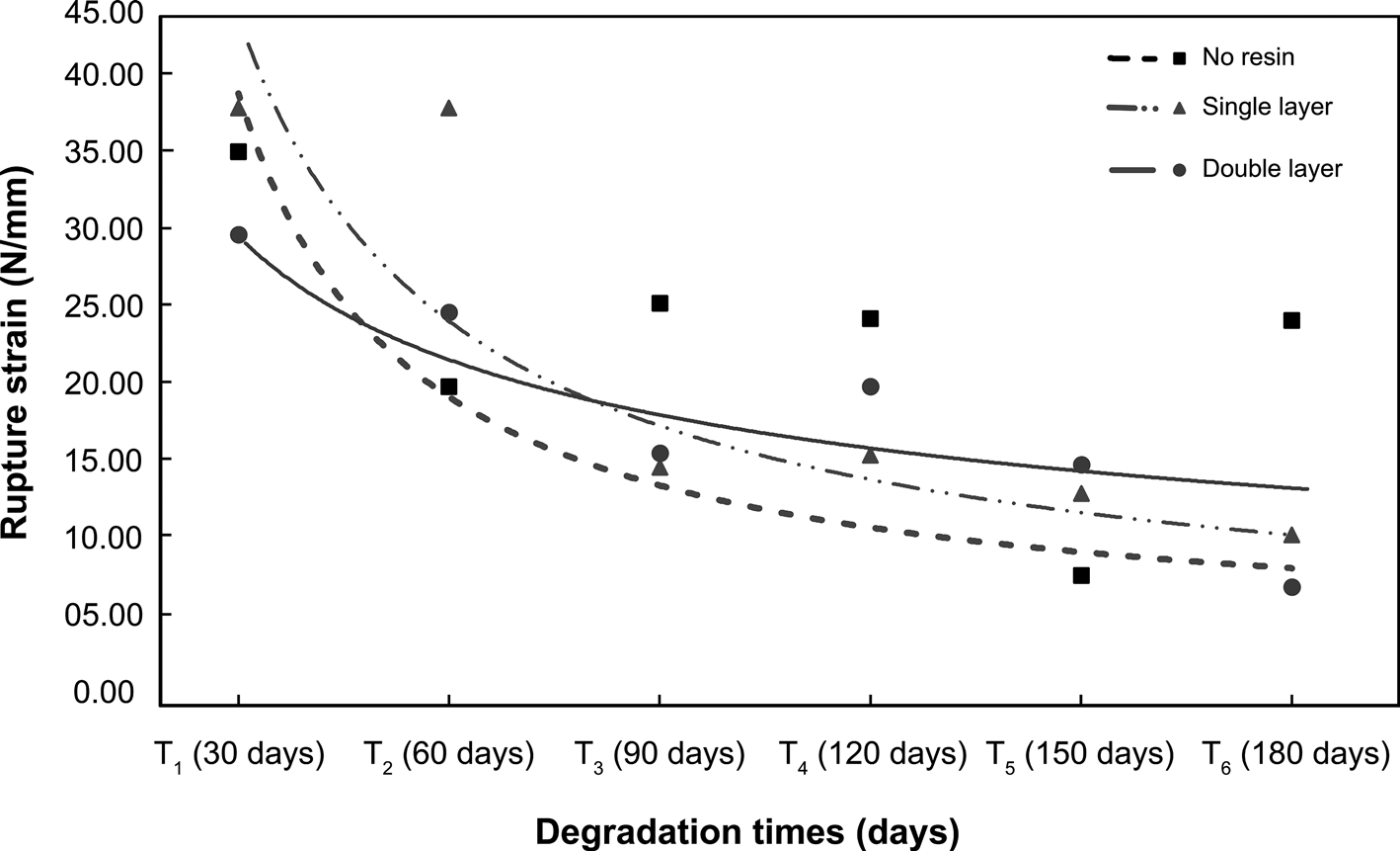
Tensile strength at break (α max) of geotextiles fibers over 180 days.

Figure 4 presents data from the regression tests for maximum traction (α max). Comparing independent samples of *T. domingensis* geotextile, it was observed that the losses of maximum resistance due to the exposure of the geotextile to degradation were statistically significant in the three treatments (without resin: *F* (1, 45) = 31.386, *p* < 0.001; _adjusted_ *R*^2^ = 0.411; single layer: *F* (1, 45) = 22.618, *p* < 0.001; _adjusted_ *R*^2^ = 0.334; double layer: *F* (1, 45) = 24.497, *p* < 0.001; _adjusted_ *R*^2^ = 0.338). The data demonstrates that there is a more significant influence to the loss of maximum resistance related to degradation for fibers that are not treated with waterproofing in comparison to the ones that are, a factor that may have generated a greater difference in mean at the end of the experiment for the untreated fibers.

Natural fibers show reductions in tensile strength due to increased depolymerization at higher temperatures^44^. Thus, the application of two layers of waterproofing resin may have reduced the effective temperature in the fibers as a result of the reduction in direct contact of the fibers with the heated soil during the exposure time. Das et al. observed that the amount of lignin present in coconut fibers (*C. nucifera*) reduced its resistance by 6% when exposed to 1 h of heating at 180 °C. While studying jute fibers (*Corchorus capsularis*), Khan et al^45^. noted that fibers exposed to degradation lost 10% of tensile strength when exposed to 170 °C in 1 h since, as the applied temperature increases, there is an increase in creep deformations and a decrease in elastic stiffness in geotextiles non-woven^46^.

The regression coefficient *B* for the treatment without waterproofing resin indicates that every 30 days of degradation, the *T. domingensis* fiber tensile strength decreases by 13.86% (*B* = − 14.446 N/mm^−1^, 95% [BCa = − 19.493–− 9.252]). This amount is reduced by 11% with the application of a double layer (*B* = − 12.408 N/mm^−1^, 95% [BCa = − 17.057–− 7.359]). Therefore, the treatment with the application of 2 layers of waterproofing resin plays an important role in controlling the residual losses of the tensile strength of this geotextile.

Differences in the tensile strength of the control sample (without waterproofing resin) and the geotextile treated with the resin were also analyzed through cross-sectional statistics between periods.

To understand the best influenced period and the differentiation of treatments' tensile strength losses, post hoc tests were carried out using each treatment longitudinally. The initial time (T_1_) was considered the control data for each treatment. The results show that the most stable significant differentiation of the natural degradation of the fibers between the treatments occurred after a 150-day exposure (T_5_). The geotextile was subjected to a 90-day exposure time.

During a 150-day period, the control treatment showed an average tensile strength of 1.899 N/mm^−1^ and residual strength of R _RES_ = 21.66% (ΔM = 6.822 N/mm^−1^; *p* < 0.001, Cohen's D = 1.530); however, when the geotextile was treated with one layer of waterproofing resin, the fibers presented an average of 2.982 N/mm^−1^ and R_RES_ = 33.66% for the same time (ΔM = 5.871 N/mm^−1^; *p* < 0.001, Cohen’s D = 0.606), both showing significant differences.

When the geotextile was submitted to a double waterproofing layer during the same period (T_5_), the fibers showed marginally significant differences in tensile strength and lower power of effect than the other treatments (ΔM = 5.556 *p* < 0.055, Cohen’s D = 0.488), with a mean of 4.373 N/mm^−1^. Residual strength values higher than the other studied treatments were also noted R_RES_ = 55.05% (Table 1).

**Table 1:** Tensile strength of geotextile fibers from *T. domingensis* over 180 days of exposure to natural degradation.

Exposure time	Treatment	Difference in means	95% CI for mean difference		Cohen's D	Pbonf
			Bottom	Highest		
T _2_ (60 days)	Without waterproofing resin	3.141	1.748	0.063	0.432	0.063
	Single layer	0.868	− 1.763	3.218	− 1.700	0.508
	Double layer	1.206	− 1.598	4.441	− 0.095	0.447
T _3_ (90 days)	Without waterproofing resin	3.085	1.906	0.105	0.449	1.000
	Single layer	4.072*	2.275	6.069	0.285	0.001
	Double layer	3.432*	0.985	6.542	0.548	0.019
T _4_ (120 days)	Without waterproofing resin	1.993*	2.058	0.324	0.132	0.052
	Single layer	4.296**	2.349	6.252	0.292	< 0.001
	Double layer	2.791	0.029	5.851	0.363	0.063
T _5_ (150 days)	Without waterproofing resin	6.822**	4.616	9.12	1.530	< 0.001
	Single layer	5.871**	4.282	7.628	0.606	< 0.001
	Double layer	3.566*	1.064	6.678	0.488	0.055
T _6_ (180 days)	Without waterproofing resin	7.755**	1.167	0.001	1.800	< 0.001
	Single layer	6.702**	5.191	8.417	0.633	< 0.001
	Double layer	6.912**	4.612	9.802	1.555	< 0.001

* Mean difference is significant at the 0.05 level; ** Mean difference is significant at less than 0.01.

Similar values of maximum tensile strength were also observed by Basu et al^9^. for the same period when analyzing geotextiles made from natural fibers reinforced by synthetic fibers. The obtained data demonstrate that the application of two layers of waterproofing resin extends the tensile strength and consequently improves the resistance to friction against the soil, improving the rigidity and textile structure of the blanket^47^. Carneiro et al^48^. observed in their study that the successive exposure of non-woven geotextiles to thermal oxidation without protection caused a decrease in tensile strength of up to 30.3%.

The loss of tensile strength (α max) occurs gradually in geotextiles manufactured from natural fibers^49^. One of the reasons behind the extending of resistance due to the use of protectors or waterproofing agents can be attributed to the reduction in the porosity of the material, which reduces the water infiltration, thus hindering microbial degradation. Pore reduction allows for the prolonged maintenance of key geotechnical properties^50^.

**Figure 5:**
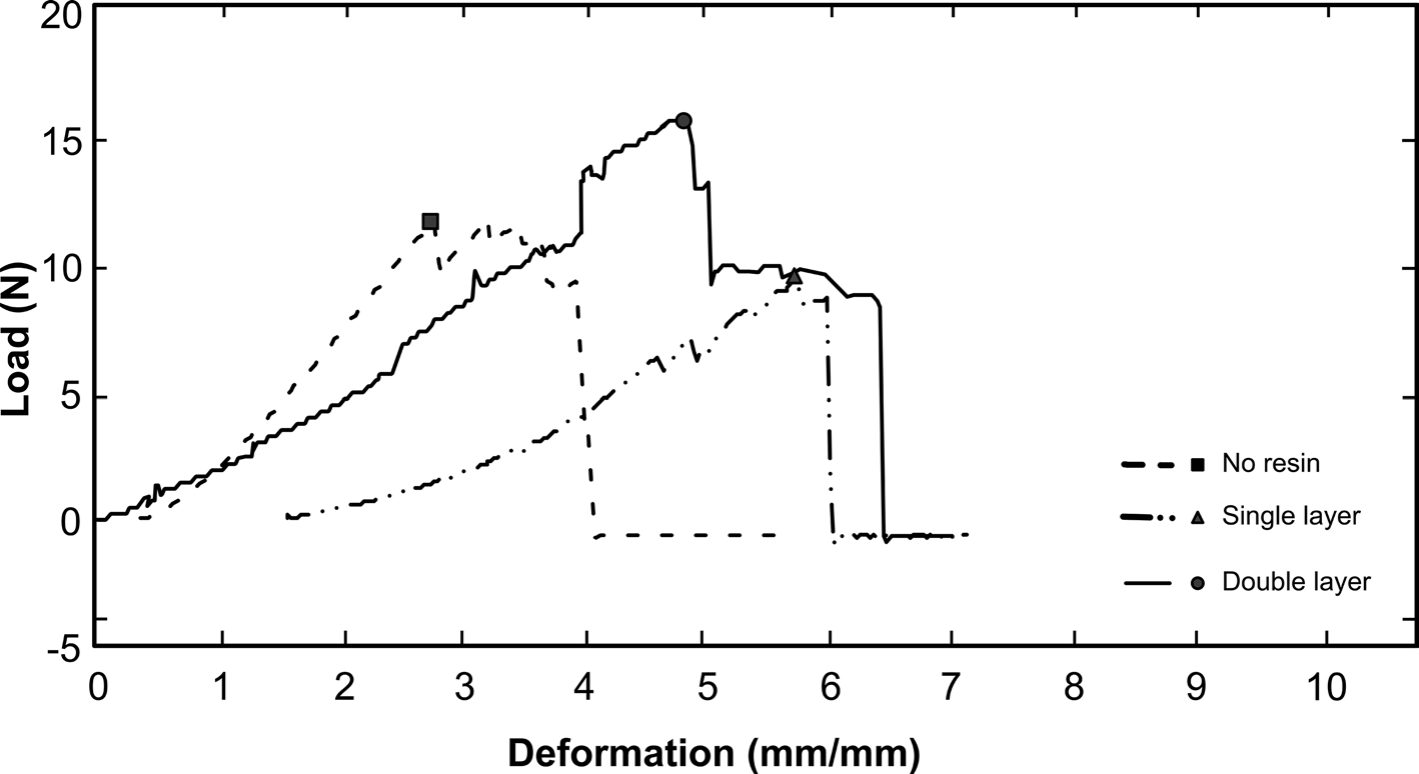
Load x. deformation for *T. domingensis* fiber samples reinforced with fiber reinforced single and double layers of waterproofing resin.

The stress–strain curve depicted in Fig. 5 illustrates a decline in tensile strength (α max) in the end of the sampling time (180 days). Such response is typical of materials that exhibit elastic behavior, such as natural fibers. For the untreated fiber, linearity is maintained until a yield limit is reached, which in this work is approximately 13.42 M. Beyond this point, the material no longer deforms elastically but enters a phase of plastic deformation, marking the onset of permanent deformation—a phenomenon similar to that observed by Leão et al^51^.

From this yield point, the stress–strain curve begins to exhibit a decline in resistance, thereby signaling the degradation of load-bearing support. The inflection point, approximately at 13.5 M, signifies a remarkable shift in stiffness, wherein a knee in the curve demarcates the transition from elastic to plastic behavior^52^. The post-yield resistance drop may be correlated with the initiation of fiber failure processes, initially through the formation and propagation of microcracks within the material, ultimately leading to fiber rupture. This is characterized by a reduced capacity of the material to bear additional loads, ending in a lessened slope of the stress–strain curve.

In comparison, *T. domingensis* fibers treated with a double layer present a superior inflection point, at 16.58 M, and those treated with a single layer exhibit an inflection point of 10.25 N. This indicates that the applied treatments might have enhanced the mechanical strength and structural stability of the fibers. Such reinforced behavior could be related to the treatment's effectiveness in shielding the fibers from environmental degradation factors, as well as in fostering improved fiber-matrix adhesion and interaction.

This durability can be attributed to the creation of a superficial coating in the geotextiles by the resin application, reducing the hydromorphic nature of the fiber, making it firmer and resulting in greater durability^53^. Another benefit obtained by resin coating is resistance to the occurrence of damage caused by granulometric degradation that results from contact with soil particles such as silt and sand^54^.

### Secant stiffness (J_sec_)

**Figure 6:**
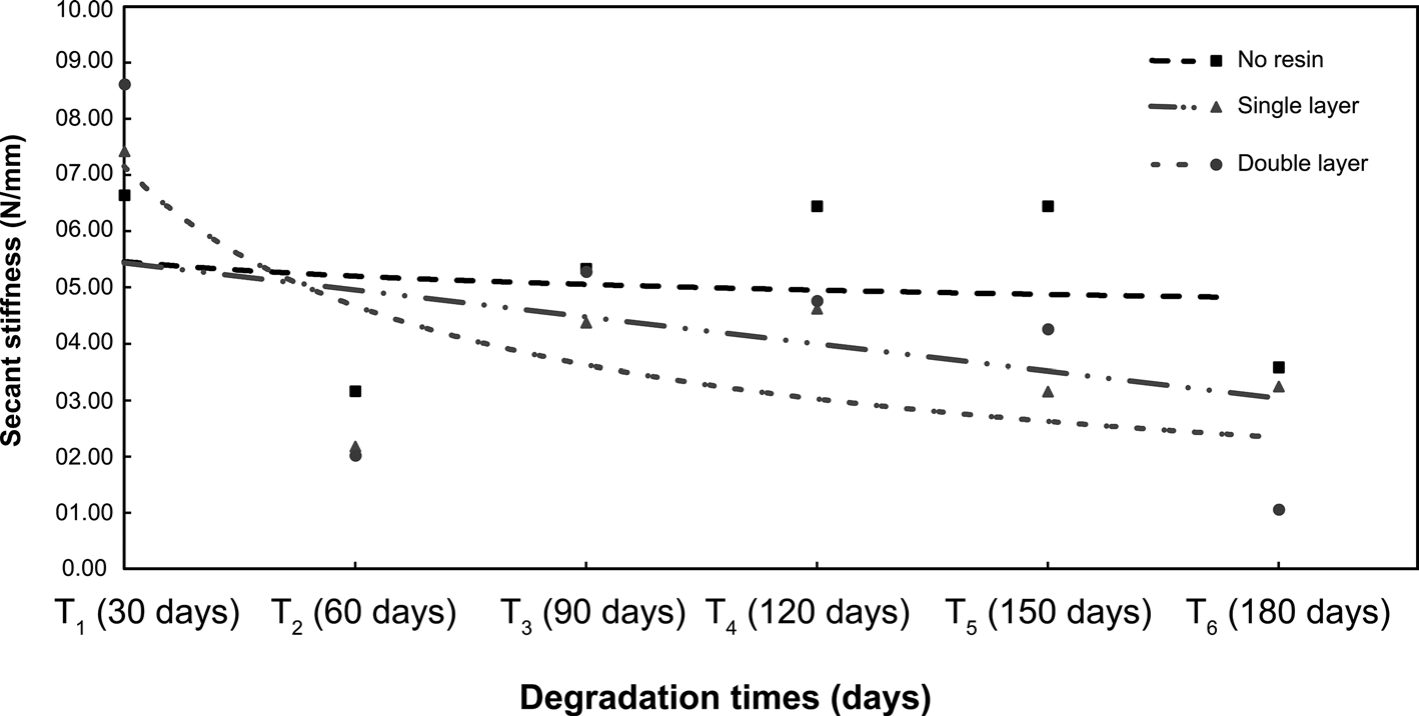
Secant stiffness (Jsec) of geotextiles over a 180-day period.

For secant stiffness (J_sec_), tests of normality distribution of residues showed that treatments T _2 (double layer)_ [KS = 0.319, *p* < 0.030; SW = 0.761, *p* < 0.016] and T_3(double layer)_ [KS = 0.318, *p* < 0.031; SW = 0.794, *p* < 0.035] did not show normal distribution. Levene’s test demonstrated that the data did not show homogeneity of variance from time T_6(120 days)_. Mauchly’s sphericity test complied with the sphericity assumption (Mauchly's *W* = 0.138 c^2^ (20) = 31.255, *p* = 0.056). The general result of the ANOVA-MR showed statistically significant differences in tensile strength scores over the proposed degradation time (*F* (5.478, 1095.73) = 36.896, *p* < 0.001; η2 = 0.672). In the post hoc longitudinal analyses (Table 2), considering the control samples as the waterproofing resin repetitions for time T_1,_ it was noticed that the secant stiffness decreased after 60 days (T_2_), with significant differences for all the treatments. In Fig. 6, it was visualized the behavior of the secant stiffness in relation to the treatments along the degradation period.

**Table 2:** Drying stiffness of fibers over 180 days of exposure to degradation.

Period of exposure to degradation	Treatments	Difference in means	95% CI for mean difference		Cohen's D	Pbonf
			Bottom	Highest		
T _2_ (60 days)	Without waterproofing resin	3.482*	0.632	6.332	2029	0.009
	Single layer	5.249**	2.399	8.098	2.304	< 0.001
	double layer	6.598**	3.748	9.448	2.897	< 0.001
T _3_ (90 days)	Without waterproofing resin	1.306	− 2.968	5.58	0.573	1.000
	Single layer	3.049	2.399	8.098	1.339	0.402
	Double layer	3.34	3.748	9.448	1.466	0.249
T _4_ (120 days)	Without waterproofing resin	0.198	− 3.807	4.204	0.087	1.000
	Single layer	2.803	− 1.203	6.808	1.231	0.442
	Double layer	3.859	− 0.147	7.864	1.694	0.066
T _5_ (150 days)	Without waterproofing resin	0.198	− 3.8	4.197	0.087	1.000
	Single layer	4.273*	0.275	8.271	1876	0.032
	Double layer	4.357*	0.359	8.355	1913	0.025
T _6_ (180 days)	Waterproofing resin	3.062	− 0.153	6.277	1.344	0.071
	Single layer	4.179*	0.964	7.394	1835	0.005
	Double layer	7.560**	4.345	10.776	3.320	< 0.001

*Average difference is significant at the 0.05 level; ** Mean difference is significant at less than 0.01.

From the 120-day exposure to degradation (T_5_), the untreated geotextile samples demonstrated a better performance compared to the other treatments–even if not that expressive when compared to the effect in the T_4 treatment_ (Average = 6.44%; ΔM = 0.198; *p* = 1.000, Cohen’s D = 0.087).

This research is distinguished in the field for its innovative incorporation of *T. domingensis* fibers and environmentally friendly resin to enhance geotextile durability. Our study’s unique approach lies in the precise selection of *T. domingensis* species widely found in many environments around the world without any other registered study related to geotextile manufacturing. Also the application of waterproofing resins, with a strong emphasis on both ecological compatibility and efficiency seems to be another highlight.

This dual commitment to sustainability and technological innovation in geotextile production sets our work apart in the areas of soil stabilization and slopes erosion control.

### Tensile deformation

The deformation of the geotextile fiber subjected to tension (ε^ii^) at times T_2 (30 days)_ [KS = 0.303, *p* < 0.051; SW = 0.884, *p* < 0.246] and T _4 (90 days)_ [KS = 0.319, *p* < 0.030; SW = 0.821, *p* < 0.146], as well as treatment with two layers of waterproofing resin at times T_4 (90 days)_ [KS = 0.303, *p* < 0.037; SW = 0.805, *p* < 0.246], did not show a normal distribution. Levene's test for tensile deformation (ε^ii^) showed that all groups showed homogeneity in variance. Mauchly’s sphericity test did not meet the sphericity assumption (Mauchly’s *W* = 0.104 C^2^ (20) = 35.758, *p* = 0.018).

**Figure 7:**
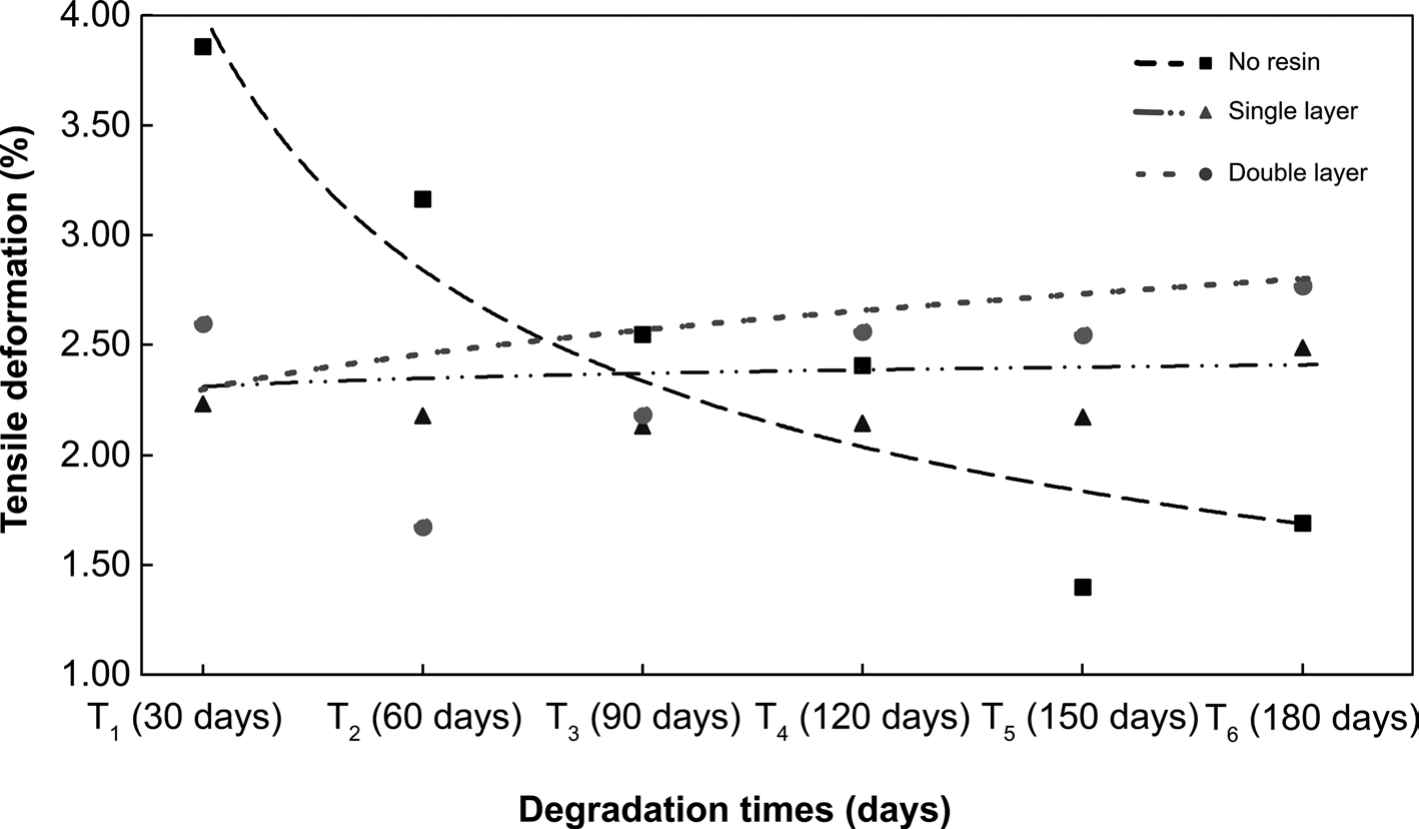
Tensile deformation (ε^ii)^ of *T. domingensis* fiber over a 180-day period for the three waterproofing resin treatments.

The overall result of the ANOVA-MR shows that there was a marginally significant difference with low power of effect in tensile deformation scores (ε^ii^) over time (*F* (5, 80,000) = 3.025, *p* < 0.041; η^2^ = 0.061). Such results demonstrate a low within-group longitudinal variability for the analyzed treatments. However, in the analysis of variance between treatments longitudinally, the difference was significant with a strong effect (*F* (10, 80,000) = 291.706, *p* < 0.004; η^2^ = 0.714). Figure 7 shows the behavior of the three treatments over a 180-day period in which the geotextile were under degradation.

Table 3 presents the tensile outcome resulted from the fiber tests. For the deformation data, the treatments with waterproofing resin do not present a significant difference during the field exposure time to degradation. The residual deformation for the samples without waterproofing treatment after a 180-day exposure period was of approximately 21.2%. In the geotextile treated with a layer of waterproofing resin, there was an increase of approximately 9.32%; while for two layers, there was an increase of 24% in deformability.

**Table 3:** Tensile deformation of fibers over a 180 − day period of exposure to degradation.

Period of exposure to degradation	Treatments	Difference in means	95% CI for mean difference		Cohen's D	Pbonf
			Bottom	Highest		
T _2_ (60 days)	Without waterproofing resin	0.275	− 1.517	2.067	0.306	1.000
	Single layer	0.097	− 0.013	3.51	0.107	1.000
	Double layer	1.244	− 0.876	3.364	1.383	1.000
T _3_ (90 days)	Without waterproofing resin	0.89	− 1.2	2.98	0.990	1.000
	Single layer	0.142	− 1948	2.232	0.158	1.000
	Double layer	0.213	− 2.26	2.686	0.237	0.906
T _4_ (120 days)	Without waterproofing resin	1.033	− 0.822	2.887	1.149	1.000
	Single layer	0.13	− 1.724	1985	0.145	1.000
	Double layer	− 0.061	− 2.255	2.134	− 0.067	1.000
T _5_ (150 days)	Without waterproofing resin	2.038*	0.657	3.419	2.267	0.002
	Single layer	0.104	− 1.277	1.485	0.116	1.000
	Double layer	0.092	− 1.542	1.726	0.102	1.000
T _6_ (180 days)	Without waterproofing resin	1.749*	− 0.013	3.51	1945	0.053
	Single layer	1.244	− 1973	1.55	0.651	1.000
	Double layer	− 0.626	− 2.71	1.458	− 0.697	1.000

*Average difference is significant at the 0.05 level.

The obtained data suggests that treatments for both one and two layers of waterproofing resin do not present significant losses in geotextile deformability in comparison with the untreated geotextile—a similar behavior to that noted by Matheus et al^55^. with manufactured-type geotextiles. This behavior indicates a slower process of structural alteration due to the loss of lignin and alteration of the degree of cellulose crystallinity in the treated samples.

Long-term changes may result in an inverse effect of reducing the values of the mechanical properties of the material if the material is more crystalline, thus, the less empty space will be present and lower the deformability Kakroodi et al^56^. Plant fibers used in the production of geotextiles have usage potential due to them having a good combination of mechanical and environmental properties^57^, but due to structural issues, they require associated protective techniques for a good adaptation of technical aspects and resilience in the field.

The geotextile derived from *T. domingensis* fibers exhibited favorable performance related to its resistance during the period of field exposure caused by climatic variables. The application of a double-layer waterproofing resin has proven effective in delaying fiber degradation for up to 180 days under exposure to climatic variables, excluding temperature^58^. This extended durability of 180 days aligns well with use in agricultural lands, especially considering the typical crop planting cycles.

This ensures that the geotextile remains effective throughout a complete planting cycle, avoiding interference with mechanized farming processes that could be hindered by more enduring fiber residues in the soil^59^. Consequently, with appropriate maintenance and treatment, *T. domingensis* geotextiles can provide a sustainable and advantageous solution in soil bioengineering applications, particularly in agricultural settings.

The *T. domingensis* in the manufacturing of geotextiles presents itself as an economical alternative to synthetic manufactured geotextiles. Regarding to its availability and the low cost of processing, geotextiles made from *T. domingensis* offer an affordable solution for soil stabilization, particularly in regions where the plant is abundant. Besides, the potential for treatment using dual crystallization waterproofing technologies emerges as a cost-effective option, enhancing the material's durability, which contributes to the economic viability of this approach^60,61^.

Regarding carbon emissions, the use of environmentally friendly resins can be part of a broader strategy to reduce the carbon footprint in construction and land stabilization projects. As seen in the study by Ashfaq et al^62^., the sustainable use of materials in construction projects, including the utilization of industrial by-products like fly ash, can lead to significant savings in costs and carbon emissions. This principle can be extended to the use of *T. domingensis* fibers. Notably, the amount of resin applied is not enough to cause pollutant damage to the environment. The selection of natural fibers with a low carbon footprint can thus contribute to the overall sustainability of the project, ensuring minimal environmental impact^63^.

## Conclusion

The protective effect of the waterproofing resin has proved the good durability performance of the *T. domingensis* fibers when exposed to field degradation during a 180-day period, demonstrating that the waterproofing resin was effective in mitigating greater damages resulting from the exposure of geotextiles to degradation; and they did not generate losses already naturally obtained by this fiber.

The applied waterproofing resin creates a coating that covers the fibers, adding extra physical protection and inhibits the degradation of the cellulosic components of the fiber.

The tested natural fiber geotextile presented results close to synthetic geotextiles, with the advantage of waste absence over its uses in the field, which is an element of remarkable importance from the environmental point of view, allowing an improvement in the resistance of geotextiles to natural soil biodegradation.

The research highlights the potential of natural fibers in sustainable uses mostly related as soil bioengineering techniques and highlights the importance of ecological compatibility in their applications. The observed data relies as a reference for future research, opening new avenues for exploring sustainable solutions in soil stabilization and erosion control.

This work not only contributes with valuable information to the field, but also paves the way for innovative and sustainable actions related to the geotextile´s applications.

## Data Availability

All data generated or analysed during this study are included in this published article (and its supplementary information files).
